# Supplementary material for: Carbon mitigation potential afforded by rooftop photovoltaic in China
Source: Nat Commun. 2023 Apr 24;14:2347. doi: 10.1038/s41467-023-38079-3 (PMC10126133; doi:10.1038/s41467-023-38079-3)
Supplement: Supplementary file 1 — Supplementary Information [file 41467_2023_38079_MOESM1_ESM.pdf]

Supplementary Information

Carbon mitigation potential afforded by rooftop photovoltaic in China

Zhixin Zhang[1,2,3], Min Chen[1,4,5,6,*], Teng Zhong[1,3,5], Rui Zhu[7], Zhen Qian[3], Fan Zhang[8], Yue Yang[3], Kai Zhang[3], Paolo Santi[9], Kaicun Wang[10], Yingxia Pu[2,5,11], Lixin Tian[12,13], Guonian Lü[1,3,5,*], Jinyue Yan[14,15,*]

1 *Key Laboratory of Virtual Geographic Environment (Ministry of Education of PRC), Nanjing Normal University, Nanjing 210023, China*
2 *School of Geography and Ocean Science, Nanjing University, Nanjing 210023, China*
3 *School of Geography, Nanjing Normal University, Nanjing 210023, China*
4 *International Research Center of Big Data for Sustainable Development Goals, Beijing 100094, China*
5 *Jiangsu Center for Collaborative Innovation in Geographical Information Resource Development and Application, Nanjing 210023, China*
6 *Jiangsu Provincial Key Laboratory for NSLSCS, School of Mathematical Science, Nanjing Normal University, Nanjing 210023, China*
7 *Institute of High Performance Computing (IHPC), Agency for Science, Technology and Research (A*STAR), 1 Fusionopolis Way, Singapore 138632, Republic of Singapore*
8 *Department of Civil and Environmental Engineering, The Hong Kong University of Science and Technology, Hong Kong, China*
9 *Senseable City Laboratory, Department of Urban Studies and Planning, Massachusetts Institute of Technology, Cambridge MA 02139, USA*
10 *Sino-French Institute for Earth System Science, College of Urban and Environmental Sciences, Peking University, Beijing 100871, China*
11 *Jiangsu Provincial Key Laboratory of Geographic Information Science and Technology, Nanjing 210023, China*
12 *Research Institute of Carbon Neutralization Development, School of Mathematical Sciences, Jiangsu University, Zhenjiang 212013, China*
13 *Key Laboratory for NSLSCS, Ministry of Education, School of Mathematical Sciences, Nanjing Normal University, Nanjing 210023, China*
14 *Department of Building Environment and Energy Engineering, The Hong Kong Polytechnic University, Kowloon, Hong Kong, China*
15 *Future Energy Center, Mälardalen University, Västerås 72123, Sweden*

*Corresponding author:
Min Chen; Email- chenmin0902@njnu.edu.cn
Guonian Lü; Email- gnlu@njnu.edu.cn
Jinyue Yan; Email- jjyan@polyu.edu.hk

Supplementary Notes

Supplementary Note 1: Definition of China's regional power grid

China's power grid is geographically divided into six geographical regions: North, Northeast, East, Central, Northwest, and South. The definition of the boundaries of the six geographical regions in this study follows the division of the Ministry of Natural Resources of China (Supplementary Table 1). The six regional power grids are managed by two major groups, among which, the North, Northeast, East, Central, and Northwest power grid are affiliated with the State Grid Corporation, while the South power grid is affiliated with the Southern Power Grid Corporation. The six regional grids have substantial differences in terms of power demand, resource endowment and power structure. For example, electricity demand in the fossil resource-rich northwest region is relatively low, leading to difficulty in consuming local renewable energy; in contrast, the eastern region relies on thermal power to meet its high electricity demand, but its fossil resources are scarce[1].

Supplementary Note 2: Baseline emission factors for regional power grids in China

We measure the carbon emissions from the regional power grids based on the "2019 Baseline Emission Factors for Regional Power Grids in China[2]". The data is calculated by the Ministry of Ecology and Environment of China based on the "Emission Factor Calculation Tool for Electricity Systems[3]" reported by the Clean Development Mechanism Executive Board (CDMEB), and includes carbon emission factors for the six regional power grids in China. Compared to bottom-up emission inventories, the baseline emission factors facilitate a comprehensive and comparable assessment of the average electricity emission intensity for each power grid[4]. The operating margin (OM) factors were calculated based on the power generation, fuel type and fuel consumption of the power plants in the grids. The build margin (BM) factors were calculated based on the weighted average of the emission factors for the newly built power plants, and the weights were determined by their power generation. The data used in the calculations was mainly from China's statistical yearbooks of energy and electricity.

Supplementary Note 3: Definition of city-level administrative districts

The definition of cities involved in this study follows the city-level statistical units used in the seventh population census of China. For the study area, a total of 354 cities are included, of which 326 are prefectures, one is corps (Production and Construction Corps in Xinjiang Province), one is new area (Xiong'an in Hebei Province), one is forest area (Shennongjia in Hubei Province), four are municipalities (Beijing, Tianjin, Shanghai, Chongqing), and 21 are province-administered county-level cities (Tianmen, Qianjiang and Xiantao in Hubei Province; Jiyuan in Henan Province; Xinji and Dingzhou in Hebei Province; Wuzhishan, Baoting, Changjiang, Ledong, Dongfang, Tunchang, Wenchang, Dingan, Chengmai, Wanning, Qionghai, Lingao, Lingshui, Baisha and Qiongzhong in Hainan Province).

Supplementary Note 4: Sensitivity analysis of the rooftop area extrapolation model

A sensitivity analysis was performed to demonstrate the effectiveness of the selected explanatory variables for rooftop area prediction. Specifically, different combinations of the four explanatory variables (road length, built-up area, population size, and night light intensity) were developed to build regression models, and the model metric of each fold was recorded in Supplementary Table 4-6.

Supplementary Note 5: Performance comparison of the rooftop area extrapolation models

We compared the performance of random forest with the decision tree, k nearest neighbors, and linear regression models. The descriptions of the three selected methods are explained below. The sample data of the 86 cities were first normalized and divided, with 90% of the data used as the training data and 10% used as the test data. We used a 10-fold cross-validation and recorded the performance of these models in each fold (Supplementary Table 8).

Decision tree：

Decision tree regression is a non-parametric method that generates regression models in a tree-like topology. It divides a dataset into progressively smaller sections while simultaneously developing an associated decision tree. The ultimate output is a tree composed of decision nodes with two or more branches and leaf nodes that reflect a numerical target. The basic steps followed in this study are explained below:

(1) Select the optimal slice variables and values. Iterate over all explanatory variables, minimize the squared error loss sweat, and determine the optimal division value.

(2) Divide the feature space and determines the output value. Based on the optimal division value, the explanatory variables are divided into range domains to obtain different feature spaces.

(3) Repeat the above steps to generate a decision tree.

K nearest neighbors:

K nearest neighbors regression is also a non-parametric technique that predicts continuous value by averaging samples from the same neighborhood. The neighborhood size is selected by cross-validation test in this study in an effort to reduce the squared error. The basic steps followed in this study are explained below:

(1) Determine the value of k and the distance definition. The value of k is selected by 10-fold cross-validation in this study, and the Euclidean distance is selected in this study.

(2) Search for the k-nearest samples. Utilizing the provided hyper-parameters, KD-tree algorithm is adopted to efficiently search numerous samples for the k-nearest samples.

(3) Averaging the value of k-nearest samples. The anticipated result of input data is the average value of k samples from the same neighborhood.

Linear Regression:

Linear regression is a regression analysis method that uses a least square function to model the relationship between one or more independent and dependent variables. The basic steps followed in this study are explained below:

(1) Establish the prediction function. Exploratory analysis is performed on the given data to specify the independent and dependent variables, assuming a linear relationship between the variables and giving the prediction function.

(2) Calculate the cost function. The cost function reflects the error between the predicted and actual values, and the goal of model training is to minimize the cost function.

(3) Update the function parameters. The sample data is used to obtain the parameters at the minimum of the cost function.

(4) Repeat steps 2-4 until the step of the cost function update is less than the set value or the number of iterations reaches the preset value.

Supplementary Note 6: Ground truth data for the validation of the rooftop area extrapolation

To obtain the ground truth data for the validation of the rooftop area extrapolation, we used the deep learning semantic segmentation method (applied in a previous work[5]) to extract the building rooftops from high-resolution satellite images. The components of the method are illustrated in Supplementary Fig. 1. The following steps were employed for extracting the rooftop area: (1) data preparation through spatial stratified sampling involving geospatial prior knowledge and data processing pipeline to augment the representativeness and number of samples; (2) creation of a deep learning segmentation model, which is based on an ensemble learning strategy and an improved prediction method to improve the rooftop extraction performance. The deep learning rooftop extraction model was trained based on Google Earth satellite imagery samples from 90 representative cities in China, covering a total area of 360 km^2.

Supplementary Note 7: Estimation of city-level rooftop availability conversion factors

To clarify the differences in rooftop availability at the city level, we selected four cities in China with different administrative levels (Beijing, Wuhan, Guiyang, and Yuxi, from highest to lowest administrative level) for our supplementary study. We considered four important aspects that affect rooftop availability, including building social function, geometric typology, slope & orientation, and shadow & obstacle. The calculation for each aspect is described below. Adopting the assumptions of the average case, the results showed that the conversion factor of rooftop availability at the city level ranged from 0.32 to 0.35, which is similar to the factor at the national level currently used. In developed cities with higher administrative levels, rooftop availability is lower due to the increased proportion of unavailable land with cultural heritage and public facilities properties. In addition, even adopting the lowest-case assumptions, the conversion factor is between 0.24 and 0.26.

Social function:

The social functions of buildings were determined based on the areas of interest (AOI) data from Baidu Map. The AOI data delineate the different social functions of urban land in detail. We identified the percentage of different functions by matching the function attributes of urban lands to the corresponding buildings. Due to the limitation of data coverage, only the attributes of some buildings can be directly determined, while the others are indirectly derived from this part of buildings. The original AOI properties were organized according to China's urban land classification code. The buildings were eventually classified into eight main categories (Supplementary Table 15). Among them, buildings with special functions (Roads and transportation, municipal facilities and culture & tourism) are considered unsuitable for the deployment of rooftop photovoltaics (RPVs).

Geometric typology, slope & orientation:

Geometric typology and slope & orientation need to be judged and measured manually from the satellite imagery. Due to the limited human labor, we took a sampling approach to conduct the survey. Buildings in China have different geometric typology, such as flat rooftops, butterfly rooftops, gable rooftops, hip rooftops, etc. Since the available data are not three-dimensional, some assumptions have to be made about the typology of pitched rooftops. To simplify the calculations, we assumed that all buildings with sloped rooftops have the ideal gable shape and that they have an average slope angle of 30°[12]. In addition, rooftops facing north (including north, northwest and northeast) are not considered for RPV installation due to the low solar energy acceptance (Supplementary Table 16).

shadow & obstacle

Shadow & obstacle are important factors in the analysis of rooftop availability. Shadows caused by nearby buildings and vegetation, as well as the presence of obstacles such as chimneys and elevator shafts, should be eliminated as much as possible to minimize calculation inaccuracies. Existing studies have provided different utilization factors under different assumptions. The utilization factor is described as the total available rooftop area for the PV installation divided by the total rooftop surface area. As shown in several studies, the values may range from 0.3 to 0.5[13-19]. Based on different assumptions of shadow & obstacle, we calculated the final rooftop availability conversion factors (Supplementary Table 17).

Supplementary Tables

Supplementary Table 1: Scope of China's geographical regions

Region	Geographical scope
North	Beijing, Tianjin, Hebei, Shanxi, Shandong, Inner Mongolia
Northeast	Liaoning, Jilin, Heilongjiang
East	Shanghai, Jiangsu, Zhejiang, Anhui, Fujian
Central	Henan, Hubei, Hunan, Jiangxi, Sichuan, Chongqing
Northwest	Shaanxi, Gansu, Qinghai, Ningxia, Xinjiang
South	Guangdong, Guangxi, Yunnan, Guizhou, Hainan

Supplementary Table 2: Changes in national RPV carbon mitigation potentials in 2020 with different combinations of panel efficiency and rooftop availability

Rooftop availability \ Panel efficiency	14%	16%	18%	20%	22%	24%	26%
15%	1141	1304	1466	1629	1792	1955	2118
25%	1901	2173	2444	2716	2987	3259	3530
35%	2661	3042	3422	3802	4182	4562	4943
45%	3422	3911	4399	4888	5377	5866	6355
55%	4182	4780	5377	5975	6572	7169	7767

Supplementary Table 3: Distribution of training samples for rooftop area extrapolation

Region	City	Number of cells	Rooftop area (km^2)	Region	City	Number of cells	Rooftop area (km^2)
Central	Bazhong	1447	122	North	Yinchuan	875	128
Central	Changde	2073	298	North	Yuncheng	1742	334
Central	Changsha	1342	352	North	Zibo	733	276
Central	Chengdu	1659	551	Northeast	Anshan	1212	170
Central	Chongqing	9511	1065	Northeast	Changchun	3447	360
Central	Ganzhou	4362	400	Northeast	Dandong	1941	124
Central	Hebi	262	90	Northeast	Haerbin	7553	405
Central	Jingmen	1436	160	Northeast	Jilin	3797	168
Central	Jiujiang	2182	207	Northeast	Jixi	3206	85
Central	Nanchang	817	197	Northeast	Qiqihaer	6247	236
Central	Nanchong	1449	205	Northeast	Shenyang	1735	377
Central	Pingxiang	434	96	Northeast	Songyuan	2974	138
Central	Wuhan	988	310	Northwest	Ankang	2796	86
Central	Yichang	2457	205	Northwest	Baiyin	2495	94
Central	Zhengzhou	911	410	Northwest	Haidong	1614	88
Central	Zigong	502	100	Northwest	Kelamayi	1002	18
East	Fuzhou	1371	277	Northwest	Lanzhou	1632	150
East	Hangzhou	1949	396	Northwest	Wulumuqi	1961	115
East	Hefei	1348	238	Northwest	Xian	1213	352
East	Nanjing	778	274	Northwest	Xining	948	98
East	Nanping	2956	156	Northwest	Yanan	4587	96
East	Ningbo	1201	482	Northwest	Zhangye	4925	113
East	Shanghai	930	662	Northwest	Zhongwei	1702	92
East	Suzhou	1012	631	South	Baise	3962	143
East	Tongling	352	56	South	Chaozhou	354	133
East	Wenzhou	1391	278	South	Dali	3133	225
East	Xiamen	196	113	South	Dongguan	268	346
East	Yancheng	1917	432	South	Guangzhou	800	413
North	Baotou	3663	136	South	Guigang	1158	201
North	Beijing	2143	639	South	Guiyang	900	146
North	Chifeng	11867	260	South	Haikou	262	80
North	Datong	1837	143	South	Heyuan	1711	128
North	Hnegshui	1112	369	South	Jiangmen	1051	246
North	Huhehaote	2245	171	South	Kunming	2323	221
North	Jinan	1270	422	South	Lijiang	2301	84
North	Jining	1374	437	South	Liupanshui	1095	101
North	Qingdao	1388	554	South	Naning	2399	331
North	Rizhao	663	203	South	Qinzhou	1184	140
North	Shijiazhuang	1652	654	South	Sanya	261	33
North	Taiyuan	871	144	South	Shenzhen	233	197
North	Tianjin	1555	604	South	Yulin (Guangxi Province)	1390	270
North	Tongliao	8124	236	South	Yuxi	1640	113
North	Weifang	1993	775	South	Zhaotong	2531	177

Supplementary Table 4: Sensitivity analysis of explanatory variables for the rooftop area extrapolation model based (MAE reports)

Road length	Built up ratio	Population	Night light	Fold 1	Fold 2	Fold 3	Fold 4	Fold 5	Fold 6	Fold 7	Fold 8	Fold 9	Fold 10	Final
✓				0.11	0.08	0.10	0.14	0.12	0.13	0.14	0.05	0.05	0.05	0.10(+/-) 0.02
	✓			0.08	0.05	0.08	0.14	0.09	0.10	0.11	0.03	0.03	0.02	0.07(+/-) 0.02
		✓		0.13	0.08	0.11	0.15	0.13	0.13	0.14	0.04	0.04	0.04	0.10(+/-) 0.02
			✓	0.11	0.07	0.09	0.13	0.11	0.14	0.14	0.05	0.06	0.05	0.10(+/-) 0.02
✓	✓			0.06	0.05	0.07	0.11	0.08	0.09	0.10	0.03	0.03	0.02	0.06(+/-) 0.02
✓		✓		0.10	0.07	0.09	0.13	0.11	0.11	0.12	0.04	0.04	0.03	0.08(+/-) 0.02
✓			✓	0.10	0.06	0.08	0.12	0.10	0.12	0.12	0.04	0.05	0.04	0.08(+/-) 0.02
	✓	✓		0.07	0.04	0.07	0.12	0.08	0.09	0.11	0.03	0.03	0.02	0.07(+/-) 0.02
	✓		✓	0.06	0.04	0.06	0.09	0.07	0.09	0.10	0.03	0.03	0.02	0.06(+/-) 0.01
		✓	✓	0.10	0.06	0.08	0.12	0.10	0.12	0.12	0.04	0.04	0.04	0.08(+/-) 0.02
✓	✓	✓		0.06	0.04	0.06	0.11	0.07	0.08	0.10	0.03	0.03	0.02	0.06(+/-) 0.02
✓	✓		✓	0.06	0.04	0.06	0.09	0.07	0.08	0.10	0.03	0.03	0.02	0.06(+/-) 0.01
✓		✓	✓	0.09	0.06	0.08	0.11	0.09	0.11	0.11	0.04	0.04	0.03	0.08(+/-) 0.02
	✓	✓	✓	0.06	0.04	0.06	0.09	0.07	0.08	0.10	0.03	0.03	0.02	0.06(+/-) 0.01
✓	✓	✓	✓	0.06	0.04	0.06	0.09	0.07	0.08	0.10	0.03	0.03	0.02	0.06(+/-) 0.01

Supplementary Table 5: Sensitivity analysis of explanatory variables for the rooftop area extrapolation model (RMSE reports)

Road length	Built up ratio	Population	Night light	Fold 1	Fold 2	Fold 3	Fold 4	Fold 5	Fold 6	Fold 7	Fold 8	Fold 9	Fold 10	Final
✓				0.24	0.17	0.20	0.27	0.23	0.26	0.32	0.11	0.11	0.10	0.20(+/-) 0.04
	✓			0.18	0.11	0.17	0.25	0.18	0.24	0.27	0.10	0.10	0.08	0.17(+/-) 0.03
		✓		0.26	0.16	0.21	0.28	0.24	0.28	0.34	0.13	0.11	0.10	0.21(+/-) 0.04
			✓	0.22	0.14	0.17	0.25	0.22	0.27	0.30	0.11	0.12	0.11	0.19(+/-) 0.04
✓	✓			0.15	0.10	0.13	0.21	0.15	0.20	0.25	0.08	0.08	0.06	0.14(+/-) 0.03
✓		✓		0.21	0.14	0.16	0.24	0.19	0.22	0.29	0.11	0.09	0.08	0.17(+/-) 0.03
✓			✓	0.20	0.13	0.15	0.23	0.19	0.24	0.28	0.10	0.10	0.09	0.17(+/-) 0.03
	✓	✓		0.16	0.10	0.14	0.22	0.15	0.21	0.26	0.09	0.08	0.06	0.15(+/-) 0.03
	✓		✓	0.15	0.10	0.12	0.19	0.15	0.20	0.25	0.08	0.09	0.06	0.14(+/-) 0.03
		✓	✓	0.20	0.12	0.15	0.22	0.19	0.24	0.28	0.10	0.10	0.08	0.17(+/-) 0.03
✓	✓	✓		0.15	0.09	0.12	0.20	0.15	0.19	0.25	0.08	0.08	0.06	0.14(+/-) 0.03
✓	✓		✓	0.15	0.10	0.12	0.18	0.15	0.19	0.24	0.08	0.08	0.06	0.14(+/-) 0.03
✓		✓	✓	0.19	0.12	0.14	0.22	0.17	0.22	0.27	0.09	0.09	0.08	0.16(+/-) 0.03
	✓	✓	✓	0.15	0.09	0.11	0.18	0.15	0.20	0.24	0.08	0.08	0.06	0.13(+/-) 0.03
✓	✓	✓	✓	0.14	0.09	0.11	0.18	0.14	0.19	0.24	0.08	0.08	0.06	0.13(+/-) 0.03

Supplementary Table 6: Sensitivity analysis of explanatory variables for the rooftop area extrapolation model (R^2 reports)

Road length	Built up ratio	Population	Night light	Fold 1	Fold 2	Fold 3	Fold 4	Fold 5	Fold 6	Fold 7	Fold 8	Fold 9	Fold 10	Final
✓				0.50	0.11	0.27	0.38	0.36	0.46	0.48	0.49	0.36	0.22	0.36(+/-) 0.07
	✓			0.71	0.59	0.46	0.46	0.58	0.56	0.61	0.63	0.48	0.57	0.57(+/-) 0.04
		✓		0.40	0.15	0.18	0.33	0.26	0.39	0.40	0.35	0.29	0.20	0.30(+/-) 0.05
			✓	0.55	0.37	0.42	0.46	0.40	0.41	0.53	0.53	0.16	0.13	0.40(+/-) 0.07
✓	✓			0.79	0.69	0.68	0.64	0.70	0.67	0.67	0.73	0.59	0.72	0.69(+/-) 0.03
✓		✓		0.61	0.40	0.50	0.53	0.53	0.60	0.57	0.56	0.56	0.46	0.53(+/-) 0.03
✓			✓	0.64	0.48	0.56	0.56	0.56	0.55	0.60	0.62	0.39	0.39	0.53(+/-) 0.04
	✓	✓		0.77	0.71	0.65	0.59	0.70	0.64	0.65	0.70	0.64	0.71	0.68(+/-) 0.03
	✓		✓	0.80	0.71	0.74	0.71	0.70	0.67	0.69	0.76	0.57	0.72	0.71(+/-) 0.03
		✓	✓	0.63	0.52	0.59	0.58	0.55	0.53	0.59	0.59	0.40	0.47	0.55(+/-) 0.03
✓	✓	✓		0.80	0.74	0.73	0.67	0.73	0.70	0.68	0.73	0.64	0.75	0.72(+/-) 0.02
✓	✓		✓	0.81	0.71	0.75	0.72	0.73	0.70	0.69	0.76	0.59	0.71	0.72(+/-) 0.03
✓		✓	✓	0.68	0.56	0.63	0.61	0.61	0.61	0.63	0.66	0.55	0.53	0.61(+/-) 0.02
	✓	✓	✓	0.81	0.74	0.77	0.72	0.73	0.69	0.69	0.77	0.63	0.75	0.73(+/-) 0.03
✓	✓	✓	✓	0.81	0.74	0.77	0.72	0.74	0.71	0.70	0.75	0.62	0.74	0.73(+/-) 0.03

Supplementary Table 7: Hyperparameters of rooftop area extrapolation model

Parameters	Values
The number of estimators	100
The minimum number of split samples	2
The function used to evaluate a split's quality	Squared error
The minimum number of samples at a leaf node	1
The number of features in searching for the best split	'sqrt'
Whether bootstrap samples are used	True
Whether to use out-of-bag samples	False
the number of samples to draw from input data	None

Supplementary Table 8: Performance comparison of the rooftop area extrapolation models

Model	Metric	Fold 1	Fold 2	Fold 3	Fold 4	Fold 5	Fold 6	Fold 7	Fold 8	Fold 9	Fold 10	Final
Decision tree	MAE (km^2)	0.08	0.06	0.08	0.13	0.11	0.11	0.11	0.03	0.04	0.03	0.08 (+/- 0.02)
	RMSE (km^2)	0.21	0.14	0.18	0.27	0.22	0.24	0.28	0.11	0.13	0.09	0.19 (+/- 0.04)
	R^2	0.62	0.36	0.40	0.39	0.38	0.54	0.61	0.54	0.10	0.34	0.43 (+/- 0.09)
Linear	MAE (km^2)	0.07	0.05	0.07	0.12	0.07	0.09	0.11	0.04	0.03	0.03	0.07 (+/- 0.02)
	RMSE (km^2)	0.15	0.09	0.13	0.20	0.14	0.20	0.26	0.08	0.08	0.06	0.14 (+/- 0.04)
	R^2	0.79	0.75	0.70	0.66	0.76	0.69	0.66	0.74	0.67	0.70	0.71 (+/- 0.03)
K nearest neighbors	MAE (km^2)	0.06	0.04	0.06	0.11	0.07	0.09	0.10	0.03	0.02	0.02	0.06 (+/- 0.02)
	RMSE (km^2)	0.15	0.09	0.12	0.20	0.15	0.20	0.25	0.08	0.08	0.06	0.14 (+/- 0.04)
	R^2	0.81	0.75	0.73	0.68	0.73	0.68	0.69	0.74	0.67	0.74	0.72 (+/- 0.03)
Random forest	MAE (km^2)	0.06	0.04	0.06	0.09	0.07	0.08	0.10	0.03	0.03	0.02	0.06 (+/- 0.02)
	RMSE (km^2)	0.14	0.09	0.11	0.18	0.14	0.19	0.24	0.08	0.08	0.06	0.13 (+/- 0.03)
	R^2	0.81	0.74	0.77	0.72	0.74	0.71	0.70	0.75	0.62	0.74	0.73 (+/- 0.03)

Supplementary Table 9: Details of rooftop area extrapolation validation at the city level

Region	City	Ground truth (km^2)	Prediction (km^2)	Aggregate error	Relative error
Central	Xuchang	242.22	198.64	-43.58	17.99%
Central	Zhangjiajie	68.33	64.80	-3.53	5.16%
Central	Deyang	172.90	193.22	20.32	-11.75%
East	Changzhou	275.12	266.51	-8.62	3.13%
East	Huaibei	95.78	96.96	1.18	-1.23%
East	Putian	152.14	151.63	-0.51	0.34%
North	Weihai	174.28	172.66	-1.62	0.93%
North	Yangquan	49.87	54.20	4.33	-8.68%
North	Zaozhuang	222.21	182.09	-40.12	18.05%
Northeast	Siping	121.22	127.59	6.37	-5.25%
Northeast	Dalian	356.97	399.79	42.82	-12.00%
Northeast	Liaoyang	90.65	106.77	16.12	-17.78%
Northwest	Xianyang	255.65	223.68	-31.97	12.51%
Northwest	Linxia	117.43	106.85	-10.58	9.01%
Northwest	Tongchuan	35.35	40.34	4.99	-14.12%
South	Danzhou	46.18	41.76	-4.42	9.57%
South	Shanwei	84.83	97.96	13.13	-15.47%
South	Beihai	79.58	89.62	10.03	-12.61%
Total		2640.72	2615.07	-25.65	-0.98%

Supplementary Table 10: Details of city clustering based on location conditions

rooftop area	Brown-Forsythe ANOVA test	
	F* (DFn, DFd)	154.6 (3.000, 92.45)
	P value	<0.0001
	P value summary	****
	Significant diff. among means (P < 0.05)?	Yes
	Welch's ANOVA test	
	W (DFn, DFd)	108.5 (3.000, 122.3)
	P value	<0.0001
	P value summary	****
	Significant diff. among means (P < 0.05)?	Yes
Solar radiation	Brown-Forsythe ANOVA test	
	F* (DFn, DFd)	296.2 (3.000, 257.2)
	P value	<0.0001
	P value summary	****
	Significant diff. among means (P < 0.05)?	Yes
	Welch's ANOVA test	
	W (DFn, DFd)	306.4 (3.000, 133.3)
	P value	<0.0001
	P value summary	****
	Significant diff. among means (P < 0.05)?	Yes
Grid emission	Brown-Forsythe ANOVA test	
	F* (DFn, DFd)	136.2 (3.000, 123.0)
	P value	<0.0001
	P value summary	****
	Significant diff. among means (P < 0.05)?	Yes
	Welch's ANOVA test	
	W (DFn, DFd)	385.0 (3.000, 109.6)
	P value	<0.0001
	P value summary	****
	Significant diff. among means (P < 0.05)?	Yes

Supplementary Table 11: Setting of urban land expansion scenarios

Social development pathways[6]	Predicted urban land area in 2020[6] (km^2)	Predicted urban land area in 2030[6] (km^2)	Urban land expansion rate from 2020 to 2030	Urban land expansion scenarios	Assumed rooftop area growth rate from 2020 to 2030
SSP3	111,698	121,632	9%	Low speed	9%
SSP4	115,089	127,803	11%		
SSP2	113,472	126,510	11%		
SSP1	115,344	130,671	13%		
SSP5	115,397	131,463	14%	High speed	14%

Supplementary Table 12: Efficiency correction factors of PV system

Efficiency correction factors	Value range
Module efficiency	0.95~0.98
Light utilization	0.95~0.98
Component surface contamination	0.96~0.98
Conversion efficiency	0.97~0.99
Inverter efficiency	0.97~0.985
Line loss	0.97~0.98
Transmission efficiency	0.97~0.985
PV system availability	0.95~0.98

Supplementary Table 13: Scale and performance parameters of PV panel

Scale and performance parameters of solar cells	Value
Length (mm)	2094
Width (mm)	1038
Height (mm)	35
Weight (kg)	23.5
Power output (P_{max}, W)	435
Efficiency（%）	20.01
Voltage at P_{max} (V)	40.75
Current at P_{max} (A)	10.68

Supplementary Table 14: Datasets for the assessment of RPV carbon mitigation potential

Usage	Dataset	Resolution	Producer	Source	Validation
Regression analysis of rooftop area	Rooftop area	Vectorized	NNU-SCENS Lab	https://doi.org/10.11888/Geogra.tpdc.271702	5
Regression analysis of rooftop area	Road network	Vectorized	OpenStreetMap	https://download.geofabrik.de/asia/china.html	7
Regression analysis of rooftop area	Land cover	10 m	Esri	https://www.arcgis.com/home/item.html?id=d6642f8a4f6d4685a24ae2dc0c73d4ac	8
Regression analysis of rooftop area	Population	100 m	WorldPop	https://hub.worldpop.org/geodata/listing?id=78	9
Regression analysis of rooftop area	Night light intensity	500 m	Earth Oservation Group	https://eogdata.mines.edu/products/vnl/#annual_v2	10
Power generation calculation	Solar radiation	10 km	(Feng & Wang, 2020)	https://data.tpdc.ac.cn/zh-hans/data/a82849b0-9af5-457d-8968-4471dd845f2e/	11
Carbon mitigation calculation	Grid emission	Statistics by regions	Ministry of Ecology and Environment of China	https://www.mee.gov.cn/ywgz/ydqhbh/wsqtkz/202012/t20201229_815386.shtml	2

Supplementary Table 15: Building social function statistics

City		Beijing	Wuhan	Guiyang	Yuxi
AOI coverage (%)	Buildings with AOI coverage	28.21	28.82	17.17	9.85
	Buildings without AOI coverage	71.79	71.18	82.83	90.15
	Total	100.00	100.00	100.00	100.00
Social functions (%)	Residential	48.50	50.72	50.66	33.39
	Logistics and warehouse	0.56	0.45	0.25	0.22
	Commercial and business	23.75	17.44	21.01	37.94
	Administration and public services	10.28	10.29	16.78	20.36
	Industrial, manufacturing	7.71	14.24	5.19	3.74
	Road and transportation	0.02	0.00	0.00	2.68
	Municipal utilities	0.17	0.06	0.02	0.14
	Culture and tourism	9.00	6.79	6.10	1.53
	Total	100.00	100.00	100.00	100.00

Supplementary Table 16: Building geometric typology and orientation statistics

City		Beijing	Wuhan	Guiyang	Yuxi
Rooftop area (km^2)	Sampled	3.82	2.32	1.02	0.78
	Unsampled	634.73	308.01	144.67	112.70
	Total	638.55	310.33	145.69	113.48
Rooftop orientation (%)	North (0°)	10.50	7.97	2.94	4.13
	North East (45°)	1.03	3.90	2.28	2.51
	East (90°)	1.73	5.74	3.17	4.32
	South East (135°)	2.31	6.76	3.18	5.52
	South (180°)	10.50	7.97	2.94	4.13
	South West (225°)	1.03	3.90	2.28	2.51
	West (270°)	1.73	5.74	3.17	4.32
	North West (315°)	2.31	6.76	3.18	5.52
	Flat	68.84	51.26	76.88	67.04
	Total	100.00	100.00	100.00	100.00

Supplementary Table 17: City-level rooftop availability conversion factor

City			Beijing	Wuhan	Guiyang	Yuxi
Utilization assumptions under the influence of shadow & obstacle	0.30	Rooftop availability conversion factor	0.24	0.24	0.26	0.26
	0.40		0.32	0.32	0.35	0.35
	0.50		0.40	0.40	0.44	0.44

Supplementary Figures

Supplementary Figure 1: Obtaining ground truth data for the validation of the rooftop area extrapolation (Figure from Zhang, Z., Qian, Z., Zhong, T. et al. Vectorized rooftop area data for 90 cities in China. Scientific Data 9, 1-12 (2022). https://doi.org/10.1038/s41597-022-01168-x- CC BY 4.0.)

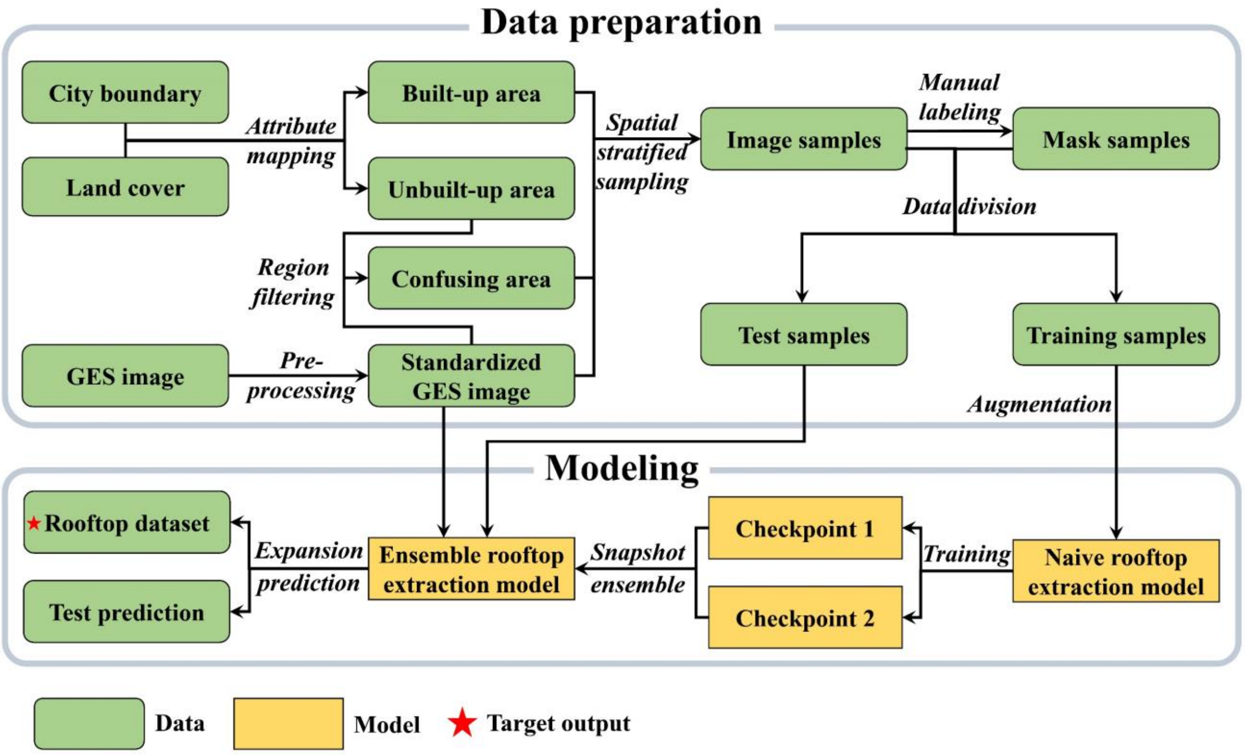

Supplementary Figure 2: Details of rooftop area extrapolation validation at the city level

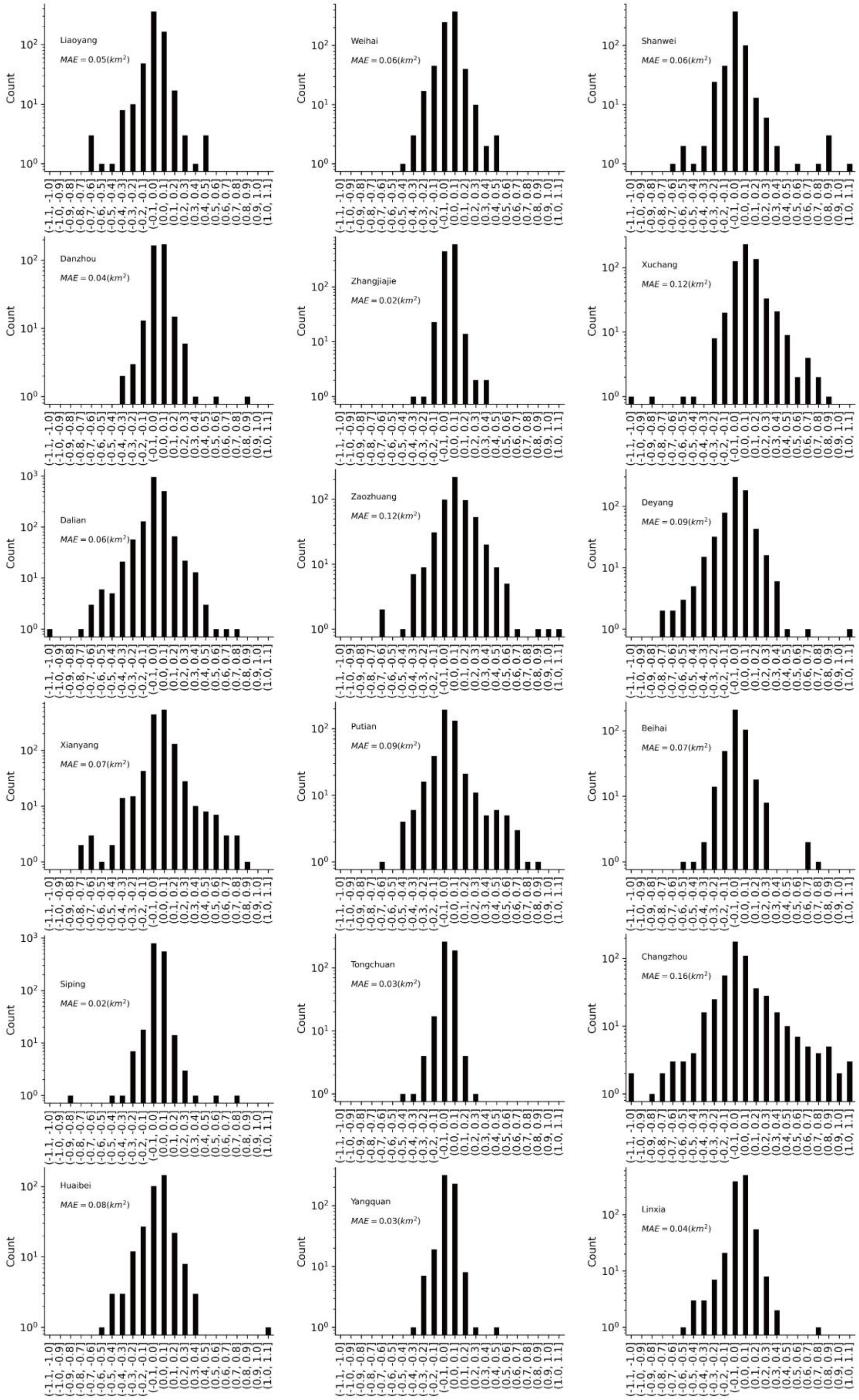

Supplementary Figure 3: RPV carbon mitigation potential per capita population. Data Credits: All the city administrative boundaries are from Amap.

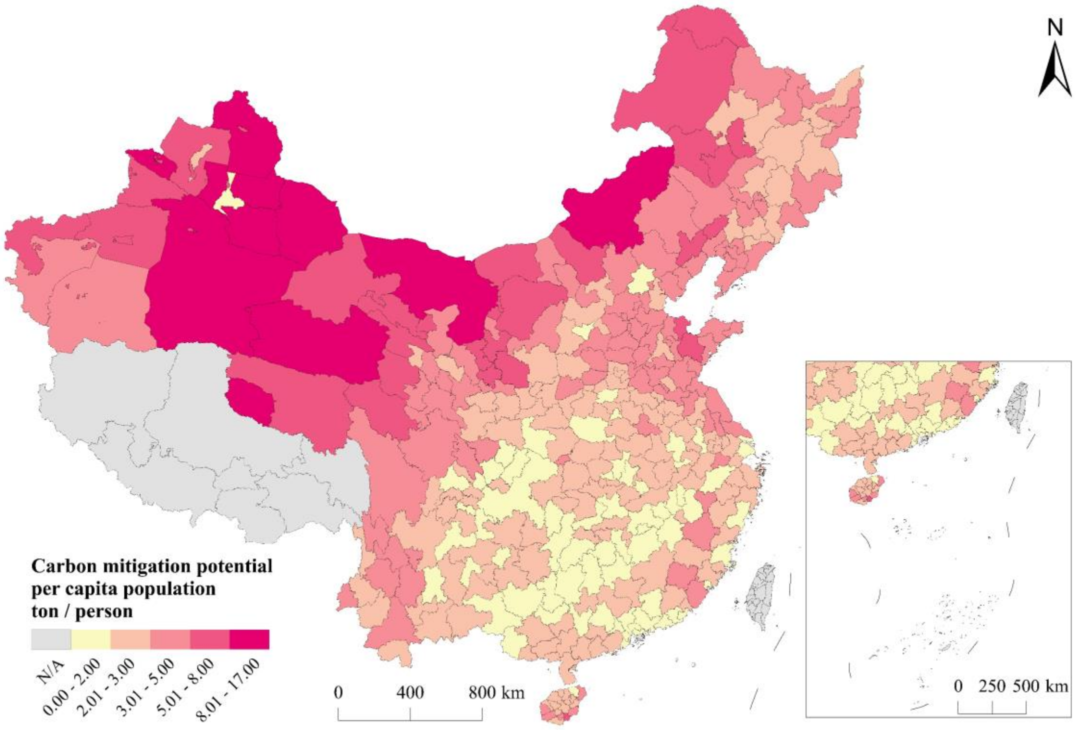

Supplementary Figure 4: RPV carbon mitigation potential per capita GDP. Data Credits: All the city administrative boundaries are from Amap.

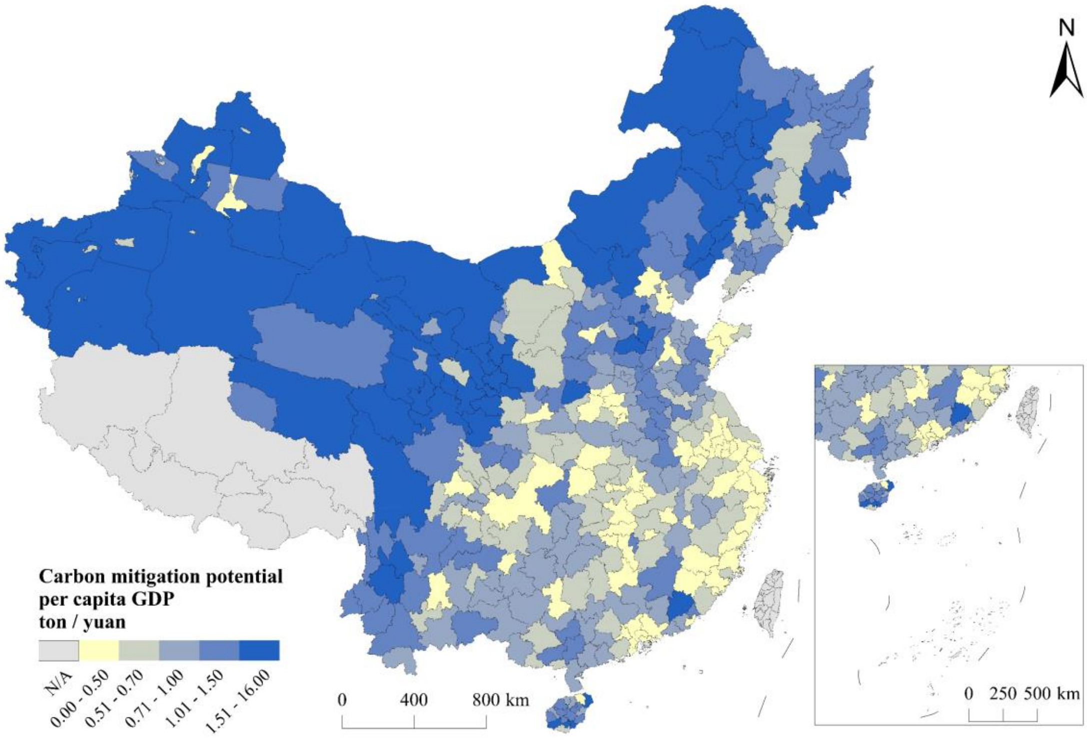

Supplementary References

1 Yi, B.-W., Xu, J.-H. & Fan, Y. Inter-regional power grid planning up to 2030 in China considering renewable energy development and regional pollutant control: A multi-region bottom-up optimization model. *Applied Energy* **184**, 641-658 (2016).

2 Ministry of Ecology and Environment of China. 2019 Baseline Emission Factors for Regional Power Grids in China (2020).

3 Clean Development Mechanism Executive Board. Tool to calculate the emission factor for an electricity system (2009).

4 Cao, C. *et al.* Incorporating health co-benefits into regional carbon emission reduction policy making: A case study of China's power sector. *Applied Energy* **253**, 113498 (2019).

5 Zhang, Z. *et al.* Vectorized rooftop area data for 90 cities in China. *Scientific Data* **9**, 1-12 (2022).

6 Chen, G. *et al.* Global projections of future urban land expansion under shared socioeconomic pathways. *Nature communications* **11**, 1-12 (2020).

7 Barrington-Leigh, C. & Millard-Ball, A. The world's user-generated road map is more than 80% complete. *PloS one* **12**, e0180698 (2017).

8 Karra, K. *et al.* in *2021 IEEE International Geoscience and Remote Sensing Symposium IGARSS.* 4704-4707 (IEEE).

9 Lloyd, C. T., Sorichetta, A. & Tatem, A. J. High resolution global gridded data for use in population studies. *Scientific data* **4**, 1-17 (2017).

10 Elvidge, C. D., Zhizhin, M., Ghosh, T., Hsu, F.-C. & Taneja, J. Annual time series of global VIIRS nighttime lights derived from monthly averages: 2012 to 2019. *Remote Sensing* **13**, 922 (2021).

11 Feng, F. & Wang, K. Merging High-Resolution Satellite Surface Radiation Data with Meteorological Sunshine Duration Observations over China from 1983 to 2017. *Remote Sensing* **13**, 602 (2021).

12 Yang, Y., Campana, P. E., Stridh, B. & Yan, J. Potential analysis of roof-mounted solar photovoltaics in Sweden. *Applied Energy* **279**, 115786, doi:https://doi.org/10.1016/j.apenergy.2020.115786 (2020).

13 Ghosh, S., Vale, R. & Vale, B. Domestic energy sustainability of different urban residential patterns: a New Zealand approach. *International Journal of Sustainable Development* **9**, 16-37 (2006).

14 Pillai, I. R. & Banerjee, R. Methodology for estimation of potential for solar water heating in a target area. *Solar Energy* **81**, 162-172 (2007).

15 Izquierdo, S., Rodrigues, M. & Fueyo, N. A method for estimating the geographical distribution of the available roof surface area for large-scale photovoltaic energy-potential evaluations. *Solar Energy* **82**, 929-939 (2008).

16 Wiginton, L., Nguyen, H. T. & Pearce, J. M. Quantifying rooftop solar photovoltaic potential for regional renewable energy policy. *Computers, Environment and Urban Systems* **34**, 345-357 (2010).

17 Vardimon, R. Assessment of the potential for distributed photovoltaic electricity production in Israel. *Renewable Energy* **36**, 591-594 (2011).

18 Yue, C.-D. & Huang, G.-R. An evaluation of domestic solar energy potential in Taiwan incorporating land use analysis. *Energy Policy* **39**, 7988-8002 (2011).

19 International Energy Agency. Potential for Building Integrated Photovoltaics. vol. 2002. (2002).
