# Supplementary material for: Carbon mitigation potential afforded by rooftop photovoltaic in China
Source: Nat Commun. 2023 Apr 24;14:2347. doi: 10.1038/s41467-023-38079-3 (PMC10126133; doi:10.1038/s41467-023-38079-3)
Supplement: Supplementary file 3 — Description of Additional Supplementary Files [file 41467_2023_38079_MOESM3_ESM.pdf]

Description of Additional Supplementary Files

File Name: Supplementary Data 1
Description: Summary of the main input and output data for city level assessments.
